# Impact of COVID-19 and Socioeconomic Factors on Delays in High-Risk MRI Breast Cancer Screening

**DOI:** 10.3390/tomography8050182

**Published:** 2022-08-27

**Authors:** Helena Teng, Wilfred Dang, Belinda Curpen

**Affiliations:** 1 Faculty of Health Sciences, McMaster University, 1200 Main Street West, Hamilton, ON L8N 3Z5, Canada; 2 Department of Medical Imaging, Sunnybrook Health Sciences, 2075 Bayview Ave, Toronto, ON M4N 3M5, Canada

**Keywords:** breast cancer screening, COVID-19, high-risk screening

## Abstract

The purpose of this study is to investigate if there was a delay in high-risk MRI breast cancer screening in our local region, if this delay is ongoing despite COVID-19 vaccinations, and if demographic and socioeconomic factors are associated with these delays. Six-hundred and sixty-five high-risk breast patients from 23 January 2018–30 September 2021 were included. Delays were determined by comparing the time in between each patients’ MRI screening exams prior to the COVID-19 pandemic to the time in between MRI screening exams during the height of the COVID-19 pandemic as well as the time in between exams when our patients started receiving vaccinations. Delays were analyzed via logistical regression with demographic and socioeconomic factors to determine if there was an association between these factors and delays. Significant time delays in between MRI screening exams were found between the pre-COVID timeframe compared to during the height of COVID. Significant time delays also persisted during the timeframe after patients started getting vaccinations. There were no associations with delays and socioeconomic or demographic factors. Significant time delays were found in between MRI high-risk breast cancer screening examinations due to the COVID-19 pandemic. These delays were not exacerbated by demographic or socioeconomic factors.

## 1. Introduction

Breast cancer continues to be the second most common cancer and accounts for approximately 25% of new cases of cancer and 13% of all cancer deaths in Canadian women [1]. After diagnosis, the probability of surviving at least five years is 88% in Canada [2,3]. Breast cancer screening is crucial for diagnosis and is recommended every 2–3 years. 

However, the COVID-19 pandemic has delayed the diagnosis of and treatment of breast cancer globally. Multiple institutions have reported the delay of routine mammographic screening through the pandemic due to institutional public health measures which led to appointment cancellations [4,5,6,7,8,9,10,11,12,13]. Multiple investigations have demonstrated that there has been a behavioral hesitancy to seek care during the first peaks of the COVID-19 pandemic, including delaying emergency care and treatment as well as delaying routine preventative care [5,6,7,8,14,15,16,17,18]. Models predicted that due to this delay, a significant increase in deaths by breast malignancy will likely result [19,20]. Additional studies have shown that delays in screening, particularly in breast care, have been also affected by socioeconomic and racial inequities [11,12,21,22,23,24].

These findings are particularly concerning and likely exacerbated in high-risk (Appendix A) patients who have underlying clinical characteristics such as genetic factors or prior history of chest radiation at a young age that predispose them to having a greater than average lifetime risk of having breast cancer [25]. These high-risk patients are subsequently screened at a younger age, with additional imaging modalities such as mammogram and magnetic resonance imaging (MRI) and more careful attention at case interpretation compared to those considered of average risk who receive only mammograms [26,27]. These high-risk patients also are more likely to present with more aggressive subtypes of interval breast malignancy at a younger age with fewer conservative treatment options, which emphasizes the need for timely breast cancer screening [28,29]. New studies identifying cancer stage migration caused by delays in diagnosis and treatment of cancer during the COVID-19 pandemic worsens this prognosis [10].

The purpose of this study is to investigate if there was a delay in high-risk breast cancer screening in our local region and if this delay is ongoing despite COVID-19 vaccination rates that are higher compared to the global population. If there are delays in high-risk breast cancer screening, then demographic and socioeconomic factors that may be associated with these delays will be identified and analyzed. A validated comprehensive area-based socioeconomic indicator tool that aggregates multiple socioeconomic factors of marginalization, used in local health policy planning, will be used for analysis. 

We hypothesize that there has been a delay in high-risk breast cancer screening due to COVID-19 and question whether more marginalized communities experience a greater impact where this is likely compounded by low vaccination rates and a higher incidence of COVID-19 cases.

The goal will be to ultimately quantify the length of screening delays of high-risk patients serviced by our institution, map these patients by their dissemination areas, and help inform future strategies to combat the deterrents to obtaining a much-needed screening breast exam. 

## 2. Materials and Methods

### 2.1. Patient Selection

This retrospective study was approved by our tertiary care Research Institute Research Ethics Board (REB # 5141). Due to the retrospective nature of the study, written or oral consent by patients was waived by the Research Ethics Board. All data was anonymized and stored according to the explicitly stated REB rules which also follow the Helsinki Declaration. 

This study was performed at a quaternary care centre with one of the largest high-risk patient referral bases in the country, with a multidisciplinary breast oncology service that includes a breast imaging team, subspecialized trained breast radiologists, and a dedicated service for patients that are at high-risk for breast malignancy. Patients who are classified as high-risk [26], enrolled in the Ontario Breast Screening Program from 23 January 2018–30 September 2021, and have had an annual high-risk screening mammogram and MRI starting from 2018 were included in our study. For all high-risk screenings, every patient that has a high-risk mammogram will have their high-risk MRI, approximately within a week of each exam. For data collection purposes, the date in which high-risk MRIs were conducted will only be used for analyses.

### 2.2. Data Retrieval 

The following data for each patient were collected from 23 January 2018–30 September 2021: date in which each high-risk screening MRI was performed, age, gender, and home postal code. All patient data was retrieved from our institutional Radiology Information System (RIS) using patient’s stated information at the time of each high-risk breast MRI exam. Data was then reviewed and manually excluded patients using each patient’s electronic health record and recent consultation notes for reference. Reasons for exclusion include: those that did not start their screening exam until after 23 January 2018, dropped out of the high-risk screening program due to geographic relocation or were removed due to advanced age (greater than 70 years old), had an interval prophylactic bilateral mastectomy, had an interval stability greater than 10 years, or did not have a scheduled annual interval screening due to clinical circumstances, such as interval breast malignancy, interval pregnancy, contrast allergy/reaction, or a non-breast related medical comorbidity that interrupted screening.

A validated aggregate area-based socioeconomic indicator tool derived from principal component factor analysis using the 2016 Canadian Census, named the 2016 Ontario Marginalization Index (Appendix B) [30], was used to determine dimensions of marginalization based on each patient’s listed home postal code. The Index is a validated tool developed using a theoretical framework based on previous work on deprivation and marginalization. It was then empirically derived using principal component factor analysis. 

Home postal codes were categorized into each patient’s respective 2016 dissemination area. Patient postal codes were matched to their respective dissemination areas using the publicly available Ontario Postal Code^OM^ Conversion File Plus (Postal Code^OM^ Conversion File Plus (PCCF+) Version 7D November 2020, Appendix C) and SAS (SAS/ACCESS^®^ 9.4 Interface to ADABAS: Reference. Cary, NC, USA: SAS Institute Inc). Postal codes identified by the PCCF+ as incomplete or non-residential were excluded. A one-to-many merge was completed with patient dissemination areas and the dissemination areas (DAUID) of the Ontario Marginalization Index 2016. These socioeconomic determinants of marginalization are categorized into residential instability, material deprivation, dependency, and ethnic concentration according to the Ontario Marginalization Index. 

The index defines each dimension as follows verbatim: Residential instability refers to area-level concentrations of people who experience high rates of family or housing instability. The indicators included in this dimension measure the types and density of residential accommodations, as well as certain family-structure characteristics. Material deprivation is closely connected to poverty, and it refers to the inability for individuals and communities to access and attain basic material needs. The indicators included in this dimension measure income, quality of housing, educational attainment, and family-structure characteristics. Dependency refers to area-level concentrations of people who do not have income from employment. It includes seniors, children, and adults whose work is not compensated. Adults included under this measure may be taking care of households, taking care of people in the community, and/or prevented from working due to disability. Ethnic Concentration refers to high area-level concentrations of people who are recent immigrants and/or people belonging to a ‘visible minority’ group (defined by Statistics Canada as “persons, other than aboriginal peoples, who are non-Caucasian in race or non-white in color”). Ordinal quintiles from this index for each dimension were used for analysis. Quintiles have been created by sorting the marginalization data into five groups, ranked from one (least marginalized) to five (most marginalized). Each group contains a fifth of the geographic units. For example, if an area has a value of five on the material deprivation scale, it means it is in the most deprived 20% of areas in Ontario. 

The time and distance travelled from the patient’s listed home address to our institution (Sunnybrook Health Sciences Centre) was determined by creating two Excel functions (=(TRAVELTIME) and =(TRAVELDISTANCE)) using a Google Maps Direction API key. The Excel functions were created using the Visual Basic Editor in Excel and a VBA-JSON convertor file. Each function utilized the patient’s postal code, Sunnybrook Health Sciences Centre’s postal code (M4N 3M5), and the API key. The first route, as determined by the API key, was used with driving as the travel mode. The output of each function was converted to minutes and kilometers as appropriate.

### 2.3. Study Design and Data Analysis

Exams were categorized by different timepoints based on the timeline of events during the COVID-19 pandemic with consideration of provincial vaccination rates, with each patient’s first 2018–2019 exam considered as a pre-pandemic control group, as the first presumptive case in the country was admitted to our institution on 23 January 2020 [31]. For the purposes of this study, exams performed in 2021 were categorized and considered as the timepoint in which there is the lowest risk of contracting COVID-19 and with least likelihood of severe disease, as this date marked the reopening of the province with a 70–80% vaccination rate amongst the vaccine eligible population within the province [32]. Exams performed in between the two aforementioned dates (in 2020) were considered to be within the height of the COVID-19 pandemic, again for the purposes of this study, as the pandemic has presented in multiple waves and is ongoing locally and globally.

The total number of high-risk screening exams were quantified and compared for each full calendar year. The times between exams for each interval exam were compared, and the delays between exams for each interval exam were quantified in days. A province-wide mandated shutdown of all screening exams, which included our institution, occurred for a period of two months, between April to June 2020 [33]. A full resumption of screening exams occurred on 1 June 2020 at our institution. The interval of time between exams between the pre-pandemic control group in between 2018–2019, ‘during COVID’ in 2020 and a possible exam timepoint in 2021, were compared using standardized *t*-tests—defining a delayed exam as one in which occurred in excess of at least 3 months (90 days) and 6 months (180 days).

Factors such as age; socioeconomic determinants of marginalization as per the Ontario Marginalization Index; and the time and distance from the patient’s home and our institution, were analyzed against the timing of interval high-risk breast imaging for correlation by logistical regression. The different degrees of association of the aforementioned factors with delayed interval screening were quantified. All statistical analyses were performed on SPSS (IBM SPSS Statistics for Windows, Version 27.0. Armonk, NY, USA: IBM Corp) with a *p* value less than 0.05 considered to be statistically significant.

## 3. Results

A total of 3569 high-risk screening breast MRI exams were performed from 23 January 2018–30 September 2021. Of the total high-risk examinations, 845 exams were completed in 2018, 1009 exams in 2019, 900 exams in 2020, and 815 exams in 2021.

Of those exams, 430 patients were excluded as these patients did not start their initial screening MRI exam until after 2018 and subsequently did not have a control study for comparison. The following patients were excluded as they had interval factors that interrupted normal screening: 26 were excluded due to interval breast cancer or having a precursor lesion to breast cancer requiring surgery; 27 were loss to follow-up, their care was transferred to another hospital or moved their care outside of the province; 7 patients who had interval diagnostic examinations that interrupted normal screening timelines; and 5 patients who had developed non-breast related comorbidities who required treatment that interrupted normal screening. Patients who had no benefit or could not have their interval MRI due to medical reasons were also included. Fifteen patients who had interval pregnancy and could not receive gadolinium for their screening breast MRI, due to potential teratogenic effects were excluded. A total of 15 patients were excluded due to interval stability for more than 10 years, having low risk due to advanced age (greater than 70 years old), or having negative genetic testing for gene mutations that increase the risk of breast cancer. Fourteen patients were also excluded due to risk-reduction bilateral mastectomies. An additional 18 patients were excluded as they did not have a postal code that corresponded to a residential address. Finally, 61 patients were excluded as they had an aborted interval screening exam due to undeclared reasons but likely due to motion, pain, or claustrophobia or had a listed undefined postal code that yielded no socioeconomic data. 

A total of 665 female patients were subsequently included in our study and analyzed. The average age at the initial exam for these patients was 48.7 (30 to 67) years. All patients live in Ontario with an average travel time of 35.3 (2.4 to 423.7) minutes and have a stated home address on average of 40.5 (0.6 to 704.8) km to our institution. The median quintiles of socioeconomic dimensions of marginalization are as follows: residential instability 2 (1 to 5), material deprivation 2 (1 to 5), dependency 2 (1 to 5), and ethnic concentration 4 (1 to 5).

The average amount of time between each patient’s first exam and second exam, the defined pre-COVID control group, was 377 with a standard deviation of 42 days. The average amount of time between each patient’s second exam and third exam, the defined group during the first few waves of COVID, was 444 with a standard deviation of 109 days. The average amount of time between each patient’s third and fourth exam, the period in which the province had started vaccinating patients, was 371 with a standard deviation of 28 days. All patients included in our study had their first, second, and third exams. Only 328/665 patients had their fourth exams during our study timeframe. 

There was a significant difference in time between each patient’s exams between the pre-COVID timepoint versus the group in the first few waves of COVID, *p* < 0.005. There was a significant difference in time between each patient’s exams between the pre-COVID timepoint versus the exams in which occurred during the period in which the province had started vaccinating patients, *p* = 0.001. 

**Figure 1:**
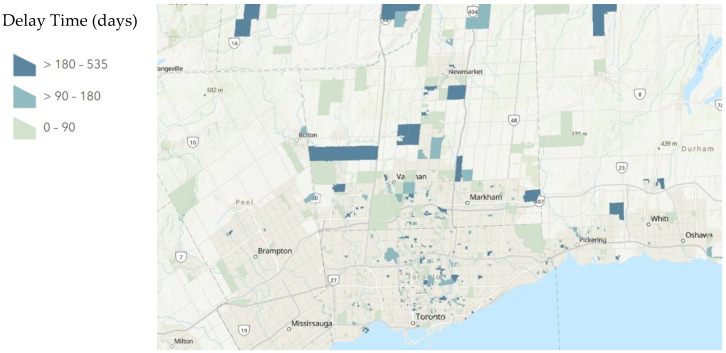
Average Delay Time of Examinations Between the pre-pandemic period (period 1) and during the height of the COVID-19 pandemic period (period 2) of Examinations Per Dissemination Area. A map of Greater Toronto Area, Ontario, Canada with patients aggregated by dissemination area and average delay time (days) calculated for each dissemination was produced by ArcGIS online. Dissemination areas of patients are marked by a gradient based on delay time. The darkest blue shows a delay in examination >180 days while the lightest blue shows a delay in examination <90 days. Dissemination areas with no color have no patient data.

**Figure 2:**
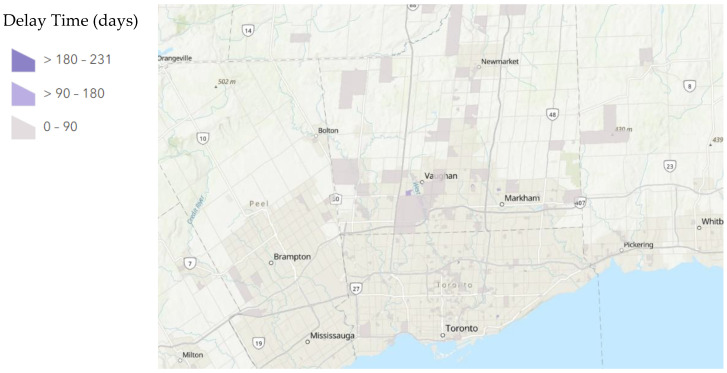
Average Delay Time of Examinations between the beginning of vaccinations period (period 3) and the height of the COVID-19 pandemic period (period 2) per Dissemination Area. A map of Greater Toronto Area, Ontario, Canada with patients aggregated by dissemination area and average delay time (days) calculated for each dissemination was produced by ArcGIS online. Dissemination areas of patients are marked by a gradient based on delay time. The darkest purple shows a delay in examination >180 days while the lightest purple shows a delay in examination <90 days. Dissemination areas with no color have no patient data.

**Figure 3:**
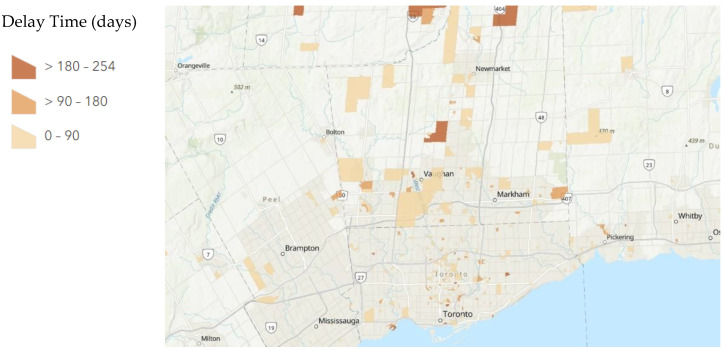
Average Delay Time of examinations between the height of the COVID-19 pandemic period (period 2) and beginning of vaccinations period (period 3) per Dissemination Area. A map of Greater Toronto Area, Ontario, Canada with patients aggregated by dissemination area and average delay time (days) calculated for each dissemination area was produced by ArcGIS online. Dissemination areas of patients are marked by a gradient based on delay time. The darkest orange shows a delay in examination >180 days while the lightest orange shows a delay in examination <90 days. Dissemination areas with no color have no patient data.

A total of 234/665 patients had a delay greater than 90 days between their first and second exams as compared to their second and third exams. A total of 2/665 patients had a delay greater than 90 days between their first and second exams as compared to their third and fourth exams. A total of 121/665 patients had a delay greater than 180 days between their first and second exams as compared to their second and third exams. A total of 2/665 patients had a delay greater than 180 days between their first and second exams as compared to their third and fourth exams. The distribution of these delays mapped to their dissemination areas is displayed below in Figure 1, Figure 2 and Figure 3.

Logistic regression analysis (Table 1, attached below) shows that no socioeconomic factors analyzed, including age (*p* = 0.553), time to travel (seconds, *p* = 0.074), distance travelled (metres, *p* = 0.085), as well as dimensions from the 2016 Ontario Marginalization Index such as residential stability (*p* = 0.573), material deprivation (*p* = 0.558), dependency (*p* = 0.402), and ethnic concentration (*p* = 0.183) have any significant contribution to delays more than 30 days between the first and second high-risk MRI exam and the second and third high-risk MRI exam.

## 4. Discussion

Our study has demonstrated a significant delay in time for high-risk breast MRI examinations between the year before COVID began compared to the year after in which COVID began. This significant delay has continued into the second year of the COVID pandemic, despite the initiation of COVID vaccinations as well as a high rate of vaccinations in our local region. The overall number of high-risk MRI examinations is similar across all years at our institution prior to excluding patients; however, this can be due to the high demand in enrolment in the high-risk program as well as the demand for MRI spots such that these time slots are filled by new patients if previously enrolled patients do not perform their exams on time. It is particularly concerning that 18.2% of patients in our included study have a time delay between exams during the height of COVID of over 180 days. It is important to note that since our institution shut down for two months (April–June 2020) during which data was collected, therefore, all patients experienced a delay of 60 days. Although, there is no determined timeframe in which a delay may lead to clinically significant breast malignancy, this delay can hypothetically increase the risk of missed interval breast cancers. Recent studies have determined that COVID-19 has led to stage migration and delays in the diagnosis and treatment of other cancers [10]. Overall, Ontario saw 41% fewer screening tests when comparing breast, lung, colon, and cervical programs in 2020. Based on historical data, this converts to 1412—1507 fewer invasive breast cancer diagnoses [34]. The impact of screening delays has been modelled. One model predicts 21,247 more cancer deaths in Canada in the next 10 years due to disruptions related to COVID-19 [35]. Specifically for breast cancer, one model predicts a 3-month (~90 day) delay in screening can lead to 310 more advanced stage diagnoses and 110 more deaths. The same model predicts that a 6-month (~180 day) delay can lead to 670 excess advanced stage diagnoses and 250 more deaths [19]. The results of this study and current literature [4,5,6,7,8,9,10,11,12,13], show how COVID-19 has led to delays in breast cancer screening. Modelling suggests delays in screening and diagnosis can lead to higher mortality rates and increase the cost of treatment for more advanced stages of cancer. Healthcare teams should work to mitigate these impacts by surveying patients who are overdue for screening and prepare for increases in advanced cancer treatments. 

There was no association between all socioeconomic factors studied and delays in high-risk screening. This may be partly explained by our healthcare system which is universal and publicly funded; potentially decreasing the impact on socioeconomic disparity by the accessibility of healthcare. This difference may also be accounted for by the differences in population groups; as our patient population was that of women who are at a high-risk of developing breast cancer versus the general population. It may be possible that high-risk women may have different attitudes and behaviours towards the necessity and priorities of breast cancer screening given the increased possibility of developing clinically significant breast cancers versus the general population. Our findings are contrasted by a study by Amram et al. (2021) which analyzed mammographic breast cancer screening in general patients in Washington State. Their study demonstrated correlations with decreased mammographic screening during the COVID-19 pandemic that were further emphasized in patients of underserved racial/ethnic groups; patients living in rural areas; and patients who self-paid for treatment or were receiving Medicaid, presumably qualifying since they are of low socioeconomic status, experienced the largest reductions in mammographic screening [23]. 

This study adds to current literature as delays in breast cancer-screening mammography have been investigated but not with particular attention to high-risk patients [4,5,6,7,8,9,10,11,12,13]. There is an emphasized importance to study these patients because of their elevated risk, poorer outcomes, as well as their need for screening with MRI. MRI is much more limited in access as compared to mammography which may compound delays in screening [27]. This study is also unique in that it investigates whether screening delays have continued in a population group with COVID-19 vaccination rates that are higher than the global average. Previous studies have only investigated mammographic screening delays in 2019 and 2020 during the height of the pandemic [4,5,6,7,8,9,10,11,12,13]. Studies in which have identified socioeconomic inequities in patients who obtain screening have also not identified factors of marginalization extensively, usually investigating age, income, insurance, and distance from a local centre as primary factors [10,12,23,29].

This investigation is limited by the scope of time in which it was performed. There are patients included who have yet to have their fourth MRI screening exam; it is unclear if this is to be scheduled further in the calendar year or may be delayed by factors of the COVID-19 pandemic. This limitation potentially confounds our comparisons between the pre-COVID control group with the post-vaccination study group as there could be further delays that are not accounted for. 

The usage of the Ontario Marginalization Index is also limited in accurately depicting current socioeconomic characteristics of the locoregional population as it based on census information from 2016. Despite this, this is the best available validated tool for socioeconomic analysis of our locoregional population and is still in use for determining current and future policy [30]. Further studies may be considered when a new index is released with the release of a new census.

Finally, confounding by the overall complexity of the COVID-19 pandemic, with regards to a high variability in timelines in new and receding waves, as well as differing case rates and vaccination rates across the region, is challenging to accurately account for. Specifically, the categorization of groups by calendar year does not account for the new and receding waves and regional policies at the time. Our study analyzes the impact of COVID-19 on MRI breast cancer-screening patients at our local institution, but differences in the degree of administrative restrictions and patient behaviours depending on case incidence and vaccination rates may differ, which may lead to differing results regarding screening delay. However, globally, current research has demonstrated an overall delay in all screening, particularly mammographic breast cancer screening in the general population [4,5,6,7,8,9,10,11,12,13].

## 5. Conclusions

We found significant delays in high-risk MRI breast cancer screening in our locoregional population over the course of the COVID-19 pandemic with a relatively high rate of patients being delayed more than 180 days for their screening exam. Despite a relative high rate of vaccinations, this significant delay has continued over the course of the pandemic. This delay has not been exacerbated by socioeconomic factors such as age, distance travelled, time to travel to our institution, residential instability, material deprivation, dependency, and ethnic concentration. Results of this study should be considered to mitigate the impact of these delays on stage migration and patient outcomes. 

## Figures and Tables

**Table 1:** Logistical regression analysis comparing socioeconomic factors versus patients with more than 30 days of delay between first and second exams (pre-COVID control) and second and third exams (during the height of COVID).

Variables	Significance	Exp(B)	95% C.I. for Exp(B)	
			Lower	Upper
Residential Instability	0.573	1.031	0.926	1.149
Material Deprivation	0.558	.957	0.827	1.108
Dependency	0.402	1.058	0.927	1.207
Ethnic Concentration	0.183	1.123	0.947	1.332
Travel Time (seconds)	0.074	1.001	1.000	1.001
Distance to Travel (metres)	0.085	1.000	1.000	1.000
Age (years)	0.553	0.994	0.975	1.014

## Data Availability

Not applicable.

## Appendix A. Definition of High-Risk Patients as per the Ontario High Risk Screening Program [26]

Ontario women ages 30 to 69 can get screened through the High Risk OBSP if they have a referral from their doctor, a valid Ontario Health Insurance Plan number, no acute breast symptoms, and fall into one of the following risk categories:

- Are known to have a gene mutation that increases their risk for breast cancer (e.g., BRCA1, BRCA2, TP53, PTEN, CDH1)
- Are first-degree relatives (parent, brother, sister or child) of someone who has a gene mutation that increases their risk for breast cancer (e.g., BRCA1, BRCA2, TP53, PTEN, CDH1), have already had genetic counselling and have chosen not to have genetic testing
- Have been assessed by a genetics clinic (using the IBIS or BOADICEA tools) as having a 25% or greater lifetime risk of breast cancer based on personal family history
- Have had radiation therapy to the chest to treat another cancer or condition (e.g., Hodgkin lymphoma) before age 30 and at least 8 years ago.

## Appendix B. Ontario Marginalization Index

Please see attached User Guide to the Ontario Marginalization Index for their definitions on their categories of marginalization [30].

## Appendix C

The Postal Code^OM^ Conversion File (PCCF) is a digital file which provides a correspondence between the Canada Post Corporation (CPC) six-character postal code^OM^ and Statistics Canada’s standard geographic areas for which census data and other statistics are produced. Through the link between postal codes^OM^ and standard geographic areas, the PCCF permits the integration of data from various sources [36].
